# Supplementary material for: Phage anti-CBASS and anti-Pycsar nucleases subvert bacterial immunity
Source: Nature. 2022 Apr 8;605(7910):522–6. doi: 10.1038/s41586-022-04716-y (PMC9117128; doi:10.1038/s41586-022-04716-y)
Supplement: Supplementary file 1 — This file contains Supplementary Fig. 1 and Tables 1 and 2. [file 41586_2022_4716_MOESM1_ESM.pdf]

nature portfolio

https://doi.org/10.1038/s41586-022-04716-y

Supplementary information

Phage anti-CBASS and anti-Pycsar nucleases subvert bacterial immunity

In the format provided by the authors and unedited

Supplementary Figure 1 | Uncropped gel and TLC images

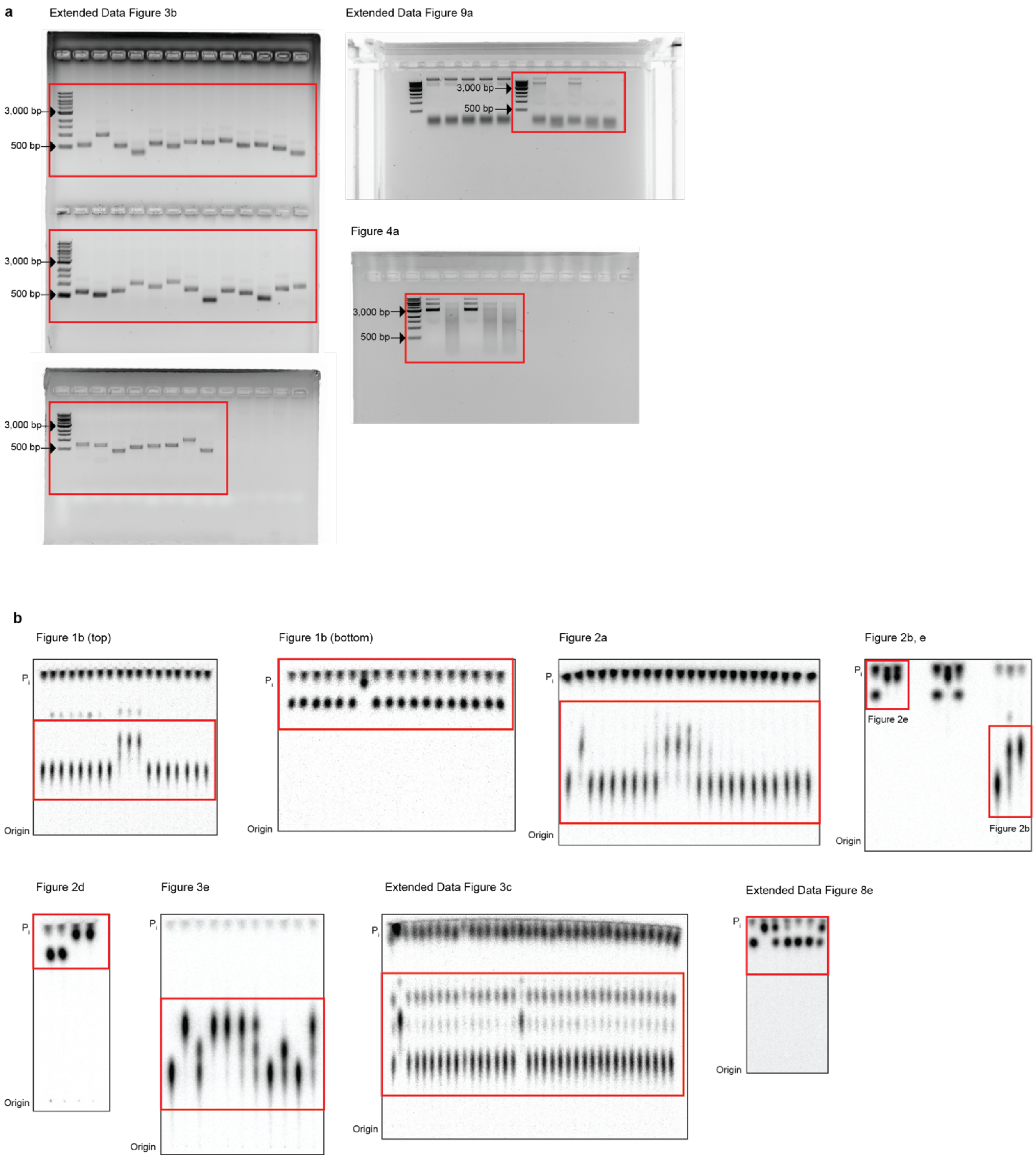

Supplementary Table 1 | Summary of phages and growth conditions used in biochemical screen

Phage	Reference	Bacterial strain	Lysate sampling time point (min)
T2	DSMZ: DSM 16352	*E. coli* MG1655	25
T4	U. Qimron	*E. coli* MG1655	25
T6	DSMZ: DSM 4622	*E. coli* MG1655	25
Escherichia phage vB_EcoM-G28	DSMZ: DSM 103876	*E. coli* MG1655	25
Escherichia phage vB_EcoM-G50	DSMZ: DSM 103881	*E. coli* MG1655	25
Escherichia phage vB_EcoM-G2540-3	DSMZ: DSM 103888	*E. coli* MG1655	25
Escherichia phage vB_EcoM-G10400	DSMZ: DSM 103894	*E. coli* MG1655	25
Escherichia phage vB_EcoM_G2540	DSMZ: DSM 103895	*E. coli* MG1655	25
Escherichia phage vB_EcoM-G2133	DSMZ: DSM 104754	*E. coli* MG1655	25
Escherichia phage vB_EcoM-G2494 (Escherichia phage RB49)	DSMZ: DSM 103886	*E. coli* MG1655	25
Escherichia phage vB_EcoM-KAW1E185	DSMZ: DSM 104099	*E. coli* MG1655	25
Escherichia phage vB_EcoM-fHo-Eco02	DSMZ: DSM 106561	*E. coli* MG1655	60
Escherichia phage vB_EcoM_MM02	DSMZ: DSM 29475	*E. coli* MG1655	25
Escherichia phage vB_EcoM-R5505	DSMZ: DSM 101797	*E. coli* MG1655	25
T5	U. Qimron	*E. coli* MG1655	25
Escherichia phage vB_EcoS-VAH1	DSMZ: DSM 103292	*E. coli* MG1655	25
Escherichia phage vB_EcoS_HdH2	DSMZ: DSM 103285	*E. coli* MG1655	25
Escherichia phage vB_EcoS_EASG3	DSMZ: DSM 103294	*E. coli* MG1655	25
Escherichia phage vB_EcoS_HASG4	DSMZ: DSM 103298	*E. coli* MG1655	25
SECphi4	Millmann et al., Cell, 2020	*E. coli* MG1655	25
SECphi6	Millmann et al., Cell, 2020	*E. coli* MG1655	25
SECphi18	Doron et al. 2018	*E. coli* MG1655	25
Escherichia phage vB_EcoS-PT-XU06	DSMZ: DSM 28622	*E. coli* MG1655	25
SECphi11	Sorek lab	*E. coli* MG1655	25
SECphi12	Sorek lab	*E. coli* MG1655	25
SECphi13	Sorek lab	*E. coli* MG1655	25
SECphi17_2	Sorek lab	*E. coli* MG1655	25
SECphi17	Doron et al. 2018	*E. coli* MG1655	45
SECphi27	Doron et al. 2018	*E. coli* MG1655	15
P1	U. Qimron	*E. coli* MG1655	60
Lambda-vir	U. Qimron	*E. coli* MG1655	45
T7	U. Qimron	*E. coli* MG1655	15
SPO1	BGSC: BGSCID 1P4	*B. subtilis* BEST7003	120
SPP1	BGSC: BGSCID 1P7	*B. subtilis* BEST7003	70
phi105	BGSC: BGSCID 1L11	*B. subtilis* BEST7003	70
SPBeta	BGSC: BGSCID 1L5	*B. subtilis* BEST7003	220
SPR	BGSC: BGSCID 1L56	*B. subtilis* BEST7003	70
SBSphiC	Doron et al., Science, 2018	*B. subtilis* BEST7003	70
SBSphiJ	Doron et al., Science, 2018	*B. subtilis* BEST7003	120
phi3T	BGSC: BGSCID 1L1	*B. subtilis* BEST7003	60
Rho11	BGSC: BGSCID 1L27	*B. subtilis* BEST7003	100
SpBetaL2	Sorek lab	*B. subtilis* BEST7003	60
SpBetaL3	Sorek lab	*B. subtilis* BEST7003	60
SpBetaL6	Sorek lab	*B. subtilis* BEST7003	50
SpBetaL7	Sorek lab	*B. subtilis* BEST7003	50
SpBetaL4	Sorek lab	*B. subtilis* BEST7003	60
SpBetaL9	Sorek lab	*B. subtilis* BEST7003	50
SpBetaL8	Sorek lab	*B. subtilis* BEST7003	120
SpBetaL5	Sorek lab	*B. subtilis* BEST7003	60
SpBetaL1	Sorek lab	*B. subtilis* BEST7003	60
SBSphiJ1	Sorek lab	*B. subtilis* BEST7003	100
SBSphiJ3	Sorek lab	*B. subtilis* BEST7003	230
SBSphiJ4	Sorek lab	*B. subtilis* BEST7003	100
SBSphiJ5B	Sorek lab	*B. subtilis* BEST7003	100
SBSphiJ5	Sorek lab	*B. subtilis* BEST7003	100
SBSphiJ6	Sorek lab	*B. subtilis* BEST7003	70
SBSphiJ7	Sorek lab	*B. subtilis* BEST7003	100

Supplementary Table 2 | Summary of data collection, phasing and refinement statistics

	Acb1 Apo **(7T26)**	Acb1–3′3′-cGAMP **(7T27)**	Acb1 SeMet	Bsp38 Apyc1 Apo **(7T28)**	*P. J14* Apyc1 SeMet **(7U2R)**	*P. xerothermodurans* Apyc1 Apo **(7U2S)**
**Data collection**						
Space group	P 4_1 2_1 2	P 4_1 2_1 2	P 4_1 2_1 2	P 3_2 2 1	P 6_1 2 2	P 1 2_1 1
Cell dimensions						
a, *b*, *c* (Å)	43.85, 43.85, 154.15	43.85, 43.85, 154.15	57.40, 70.44, 104.60	82.68, 82.68, 76.83	102.87, 102.87, 147.62	60.03, 58.29, 76.19
α, β, γ (°)	90.0, 90.0, 90.0	90.0, 90.0, 90.0	90.00, 90.00, 90.00	90.00, 90.00, 120.00	90.00, 90.00, 120.00	90.00, 101.9, 90.00
Resolution (Å)	43.19–1.15 (1.17–1.15)	43.85–1.20 (1.22–1.20)	43.52–1.24 (1.26–1.24)	38.42–2.70 (2.83–2.70)	38.14–1.85 (1.89–1.85)	38.64–1.55 (1.58–1.55)
R_{pim}	1.2 (37.7)	1.3 (60.5)	1.1 (38.8)	7.1 (73.2)	3.8 (66.6)	7.8 (29.2)
$I / \sigma(I)$	23.3 (1.4)	23.2 (1.4)	29.8 (1.8)	5.9 (1.1)	12.1 (1.2)	5.9 (1.1)
Completeness (%)	99.5	99.1 (94.9)	100 (100)	100.0 (100.0)	99.8 (99.1)	99.5 (97.6)
Redundancy	24 (12.7)	16.9 (14.5)	51.5 (50.5)	13.3 (13.5)	11.1 (11.3)	4.2 (4.2)
**Refinement**						
Resolution (Å)	43.19–1.15	42.17–1.20	–	36.26–2.68	38.14–1.85	38.64–1.55
No. reflections						
Total	1282789	813756	2245745	115438	444283	315789
Unique	53419	47844	43597	8666	39926	74391
Free	2232	2000	–	850	2000	2000
R_{work} / R_{free}	16.02 / 18.08	17.59 / 19.72	–	30.01 / 31.97	17.66 / 19.98	17.27 / 19.29
No. atoms						
Protein	1104	1135	–	1896	1937	3877
Ligand / ion	–	74	–	2 (Zn)	2 (Zn)	4 (Zn)
Water	262	220	–	36	211	690
B-factors						
Protein	18.03	12.52	–	105.68	45.05	13.71
Ligand / ion	–	13.85	–	117.52	35.64	16.31
Water	30.76	25.51	–	47.86	47.83	25.73
R.m.s. deviations						
Bond lengths (Å)	0.011	0.022	–	0.002	0.012	0.006
Bond angles (°)	1.281	2.137	–	0.484	1.177	0.927

*All datasets were collected from individual crystals. *Values in parentheses are for the highest resolution shell.
